# A large waterborne outbreak of campylobacteriosis in Norway: The need to focus on distribution system safety

**DOI:** 10.1186/1471-2334-8-128

**Published:** 2008-09-24

**Authors:** Irena Jakopanec, Katrine Borgen, Line Vold, Helge Lund, Tore Forseth, Raisa Hannula, Karin Nygård

**Affiliations:** 1 Department of Infectious Disease Epidemiology, Norwegian Institute of Public Health, PO Box 4404 Nydalen, N-0403 Oslo, Norway; 2 European Programme for Intervention Epidemiology Training (EPIET), European Centre for Disease Prevention and Control (ECDC) Stockholm, Sweden; 3 Municipal Health Authority, Røros, Norway; 4 Food Safety Authority, District office of Gauldal, Støren, Norway; 5 Department of Medical Microbiology, St. Olavs Hospital, Trondheim, Norway

## Abstract

**Background:**

On 7 May 2007 the medical officer in Røros (population 5600) reported 15 patients with gastroenteritis. Three days later he estimated hundreds being ill. Untreated tap water from a groundwater source was suspected as the vehicle and chlorination was started 11 May. *Campylobacter *was isolated from patients' stool samples. We conducted an investigation to identify the source and describe the extent of the outbreak.

**Methods:**

We undertook a retrospective cohort study among a random sample of customers of Røros and neighbouring Holtålen waterworks. Holtålen, which has a different water source, was used as a control city. We conducted telephone interviews to gather data on illness from all household members. One randomly selected household member was asked about detailed exposure history. The regional hospital laboratory tested patients' stools for enteropathogens. *Campylobacter *isolates were typed by AFLP for genetic similarity at the Norwegian Institute of Public Health. Local authorities conducted the environmental investigation.

**Results:**

We identified 105 cases among 340 individuals from Røros and Holtålen (Attack Rate = 31%). Tap water consumption was the only exposure associated with illness. Among randomly selected household members from Røros, a dose-response relationship was observed in daily consumed glasses of tap water (χ^2 ^for trend = 8.1, p = 0.004). *Campylobacter *with identical AFLP was isolated from 25 out of 26 submitted stool samples. No pathogens were detected in water samples. We identified several events that might have caused pressure fall and influx of contaminated water into the water distribution system. On two occasions, pressure fall was noticed and parts of the distribution system were outdated.

**Conclusion:**

The investigation confirmed a waterborne outbreak of campylobacteriosis in Røros. Although no single event was identified as the cause of contamination, this outbreak illustrates the vulnerability of water distribution systems. Good quality source water alone is not enough to ensure water safety. For a better risk management, more focus should be put on the distribution system security. Waterworks personnel should monitor the pressure regularly; reduce the leakage by upgrading the distribution network and use chlorination when conducting maintenance work.

## Background

Untreated groundwater is used in waterworks in many countries [1-4], including Norway.

The Norwegian drinking water regulations require two independent hygienic barriers in a drinking water supply system. Hygienic barriers are defined as "natural or man-made obstacles preventing occurrence of infectious, chemical or physical particles in the water so that they no longer represent any health risk"[5]. Most commonly used water treatments are chlorination, UV treatment, filtration, adding CO2 or alkaline substances [6]. Well protected groundwater sources may be exempt from treatment requirements in Norway due to their natural filtering through the layers in the ground. In such cases, a disinfection system should be available as a preparedness measure [5].

In Norway, groundwater is used as a source in 37% of the waterworks. These usually small waterworks supply 10% of the population, on average about 700 persons each [6]. In the 15-year period from 1988–2002, more than 30% (24/72) of the waterborne outbreaks in Norway were caused by contaminated groundwater [7].

*Campylobacter *is the most commonly reported bacterial cause of gastroenteritis in many developed countries [8] with a large economic burden [9]. Finding a specific source of infection in humans is difficult as *Campylobacter *is carried in the intestinal tract of all types of domestic livestock and many wild animals, including birds. The source of infection frequently remains unknown [10].

Sporadic cases with laboratory confirmed *Campylobacter *infection are notifiable to the Norwegian Surveillance System for Communicable Diseases [11]. *Campylobacter *is the most common bacterial gastrointestinal infection in Norway [11]. It was also the most frequently identified pathogen in all reported waterborne outbreaks (26%, 19/72) in Norway from 1988–2002, although the infective agent was not determined in almost half of the outbreaks [7]. Drinking untreated water was a leading risk factor for campylobacteriosis in a case-control study in Norway from 1999–2000 [12]. Other important risk factors include consuming undercooked poultry, unpasteurized milk, contaminated raw vegetables and fruits [9]. *Campylobacter *can survive 2–4 weeks in water, depending on the origin of the strain [13].

On Monday 7 May 2007, the municipal medical officer in Røros notified the Norwegian Institute of Public Health (NIPH) about 10 patients with gastroenteritis who had consulted Røros municipal health centre during the weekend. Røros is a small town, situated in mid-Norway. Its municipality has 5600 inhabitants. According to the medical officer, the estimated number of cases rose to a few hundreds in the three following days, and tap water was suspected to be the vehicle of infection. Five patients' stool samples were positive for *Campylobacter spp*.

The local Food Safety Authority, the Municipal Health Authority and NIPH conducted an outbreak investigation to identify the source of the outbreak and implement preventive measures.

## Methods

### Waterworks

**Figure 1:**
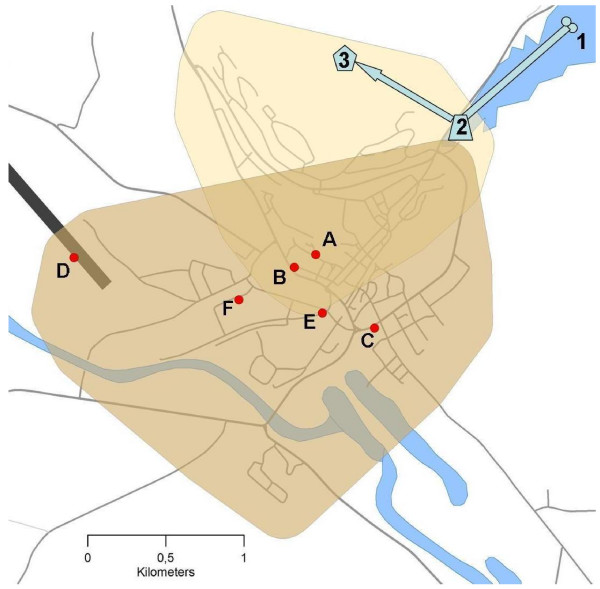
**Map of Røros with details on the waterworks and some events, which may have been relevant to the water contamination**. The higher supply zone (RH) of the waterworks is marked with yellow, the lower (RL) with brown. Parts of the waterworks (schematic): underground wells (1), common collecting tank (2) and elevated reservoir (3). Events: maintenance work 30 April (A) with closed valve (B), maintenance work 2 May (C), firemen exercise at the airport 3 and 10 May (D), low pressure observed at slaughterhouse 3 and 10 May (E) and coliform bacteria proven in a tap water sample from dairy 9 May (F).

The municipal waterworks, which supplies Røros town, provides 3600 people with tap water. Groundwater comes into the system from two wells drilled into an aquifer under an island in a lake northeast of Røros (Figure 1). Two main pipes lead the water to a common collecting tank south-west of the lake. From there, water is pumped to an elevated reservoir (900 m^3^) to provide necessary pressure for households in the **h**igher areas of Røros (R**H **zone). The **l**ower area of Røros (R**L **zone) receives water directly from the common tank, through gravity. The RH zone is mainly a residential area and the RL zone includes the town centre. The water is not chlorinated or disinfected in any way before reaching the customers.

A small neighbouring community has a separate waterworks that supplies 600 people (Holtålen waterworks).

### Pilot study

On 10 and 11 May we interviewed patients with gastrointestinal symptoms presenting at the municipal health centre in Røros using a standard trawling questionnaire. We asked them about common exposures for food and waterborne gastroenteritis.

### Retrospective cohort study

To assess water as a risk factor, we conducted a retrospective cohort study on a random sample of customers from Røros and Holtålen waterworks. We aimed at selecting 40 households from each waterworks zone (RL, RH and Holtålen). We obtained a list of all waterworks customers which we randomized using Excel. We used this list to phone the customers' households for an interview. In case of no response, the next household on the list was called.

To estimate the overall attack rate, we collected basic information from all household members: age and sex, experiencing symptoms of gastroenteritis since 1 May, duration of illness and whether or not they consulted a doctor. The date 1 May was selected based on the dates of onset reported in the pilot study. One household member from each household – the person with the nearest upcoming birthday – was selected to provide details on possible exposures during the first week in May (= "a selected household member"). The exposures included food exposures such as eating raw vegetables, dairy products and poultry; restaurants, venues, travelling, work place, amount and location of tap water consumed. We also asked whether they had noticed any tap water abnormalities such as air in the pipes or discolorations during the same period.

A case was defined as a person living in Røros or Holtålen municipality with diarrhoea (passing three or more loose stools in one day) from 1 to 14 May 2007 *OR *experiencing at least two of the following symptoms of acute gastroenteritis lasting at least two days in the same time period: nausea, vomiting, stomach cramps or pain, flatulence, blood in the stool and fever. We excluded those who recently travelled abroad, those with unknown date of gastroenteritis symptoms onset and those whose illness started before 1 May 2007 from the analysis.

The data were entered into an EpiData database and analysis was performed using Excel and Stata 9. The gender and age distribution of our cohort sample was compared with that of the general population in Røros for representativeness. We calculated water and food specific attack rates (AR), relative risks (RR), 95% confidence intervals (95% CI) and Fisher's exact p-value for the various exposures. We measured the association between the drinking water consumption and the risk of disease using the χ^2 ^for linear trend.

In agreement with the International Guidelines for Ethical Review of Epidemiological Studies by the Council for International Organisations of Medical Sciences (CIOMS) (1991), investigations of acute infectious diseases outbreaks are considered an urgent public health task in Norway under the Infectious Disease Control Act and regulations and are exempted from approval by the ethical review board.

### Retrospective study of institutions

To rapidly assess the attack rate in the different supply zones (RH and RL), we phoned and asked 11 institutions (six kindergartens and five nursing homes) about the occurrence of gastroenteritis among the employees or children/residents. We assumed children and residents in the institutions to be less mobile and therefore more likely to be exposed to tap water from only one of the two supply zones.

### Microbiological analyses of patients' stool samples

Stool samples were collected from some of the symptomatic patients at Røros and Holtålen municipal health centres. Culture and biochemical identification of bacterial enteropathogens were done according to standard microbiological methods at St. Olavs Hospital in Trondheim [14,15]. The specimens were cultured for Salmonella, Shigella and Yersinia. The specimens were further cultured for *Campylobacter *on charcoal cefoperazone desoxycholate agar (Mast Diagnostics, Merseyside, UK) in a microaerophilic atmosphere at 42°C for two days. Typical colonies including oxidase positive, hippurate positive, motile short rods were identified as *Campylobacter jejuni*. The *Campylobacter *isolates were sent to the NIPH for genotyping using amplified fragment length polymorphism (AFLP)[16,17]. The samples were also tested for adenovirus, rotavirus, norovirus, astrovirus, *Giardia lamblia *and *Clostridium difficile *toxin A/B using enzyme immuno assay or polymerase chain reaction [18,19]. Microscopy for *Cryptosporidium *oocysts and *Giardia *cysts on previously frozen samples was done at the Norwegian School of Veterinary Science.

### Environmental investigation

Waterworks personnel take weekly routine water samples from seven different locations in Røros according to their risk assessment strategy. Weekly samples are tested for total bacterial count, coliform bacteria, turbidity and colour. Quarterly samples are additionally tested for intestinal enterococci, pH and conductivity. All tests are performed according to the standard methods described in the national legislation for waterworks [20].

During the outbreak, the waterworks personnel took additional samples from different locations in the water distribution system. Samples taken on 11 May were additionally tested for Campylobacter according to the national standard method (NS-ISO 17995).

The local Food Safety Authority and the waterworks personnel thoroughly inspected the Røros waterworks, including the wells, the reservoirs and the distribution system. They assessed recent maintenance work and events which could have caused contamination.

## Results

### Epidemiology

All 15 patients included in the pilot study reported onset of gastrointestinal symptoms from May 3–7, 2007. All of them received water from Røros waterworks and drank water at home in the week before the illness onset. They did not share any other common exposures (venues, restaurants, food items etc.).

We recruited 101 households from Røros (59 from the RH zone and 42 from the RL zone) and 40 from Holtålen in the cohort study, with a total of 345 household members. Of these, five were excluded from the analysis due to unknown illness status or because the criteria for our case definition were not fulfilled. Among the 340 household members, 105 met the case definition, accounting for attack rate (AR) of 31%. The AR was 42% among customers of Røros waterworks (RH + RL) alone and 3% among customers of the neighbouring Holtdålen waterworks (Table 1).

**Table 1:** Water exposures in Røros and neighbouring Holtålen, May 2007, including all household members in the cohort

**Exposure**	**Cases**	**Total exposed**	**AR %**	**RR (95% CI)**	**Fishers exact p**
Holtålen waterworks	3	97	3	ref.	
Røros waterworks	102	243	42	13.5 (4.4 – 41.7)	< 0.00001

**Figure 2:**
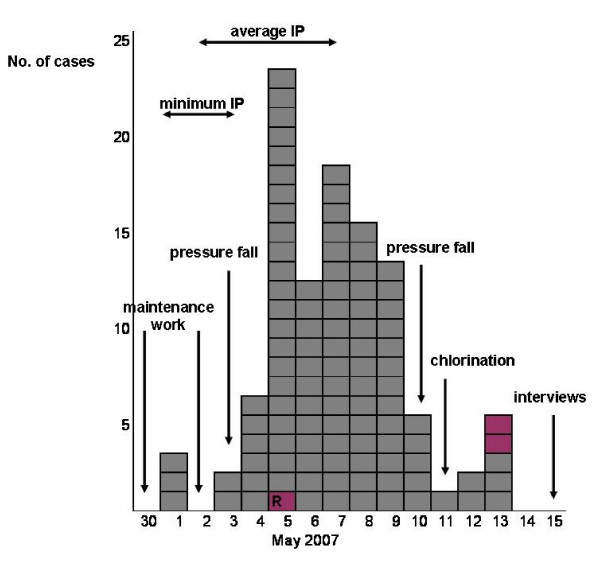
**Cases of gastroenteritis in a sample of Røros and Holtålen household members by date of illness onset (n = 105), from April 30 to May 14, 2007 and the timeline of events, which may be relevant to the water contamination**. Gray squares = Røros household members; plum squares = Holtålen household members; One case, marked with a letter R, was exposed to Røros tap water as well. IP = incubation period.

Dates of illness onset among the cases are shown in Figure 2. The curve indicates that the outbreak started between 1–3 May and peaked between 5–9 May. The median date of illness onset was 7 May.

The age and gender distribution of the household members included in the cohort were similar to the general population in Røros and Holtålen. Women represented 51% of the household members and 56% of the cases. The median age of the 340 household members was 44 years (range 0–94). The median age of the cases was 40 years (range 0–85). The majority of cases presented with diarrhoea (90%, 95/105); two of them reported blood in the stool. Among the ten cases not reporting diarrhoea, all reported stomach cramps or pain and one or more of the following symptoms: flatulence (7/10), fever (6/10), headache (3/10) and nausea (2/10). In 81 (77%) of the cases, the symptoms lasted three days or more; mean and median duration was five days (range 1–14 days). Diarrhoea (90%) and stomach cramps (77%) were the most prevalent symptoms, followed by fever (48%). Nine cases (9%) reported vomiting and six (6%) consulted a physician.

According to the Norwegian Surveillance System of Communicable diseases [11], seven patients with laboratory confirmed *Campylobacter *infection acquired in Røros during the time of the outbreak were hospitalized. A year after the outbreak the municipal medical officer reported one confirmed case of reactive arthritis in a 7 year old boy and about 10 patients with arthralgias. According to data available (Norwegian acute flaccid paralysis (AFP) surveillance database for children under 15 years old and personal communication with municipal medical officer) no diagnoses of Guillain-Barré syndrome, a rare severe acute polyneuropathy, were made. No deaths were reported as a consequence of this outbreak in The Cause of Death Register.

Only selected household members (one per household) were asked about detailed exposures, therefore 101 persons provided these details. Water consumption among selected household members in the first week of May is shown in Table 2. Five of the 101 selected household members did not drink tap water and were not ill. None of the selected household members from the RL zone drank water from the RH zone, however many selected household members living in the RH zone additionally drank water from the RL zone. The AR % in RH and RL was similar (Table 2).

**Table 2:** Attack rates (AR) by tap water drinking exposures among 101 randomly selected household members from Røros

**Tap water drinking from**	**Cases**	**Total exposed**	**AR %**	**RR (95% CI)**
no tap water	0	5	0	/

RH only	10	27	37	Ref.
RL only	20	40	50	1.3 (0.7–2.4)
both zones	17	29	59	1.6 (0.9–2.8)

RH: higher zone, RL: lower zone of Røros waterworks

**Figure 3:**
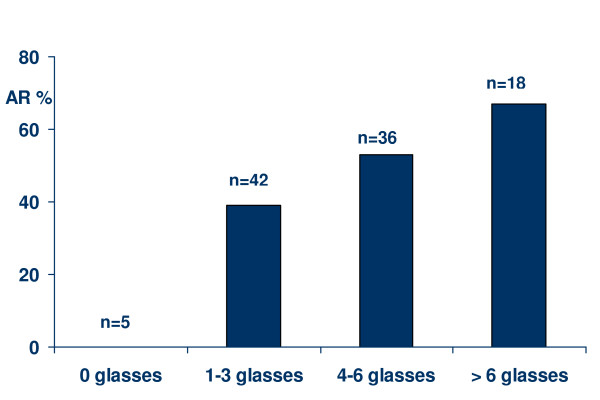
**Attack rate according to the amount of tap water consumed (glasses per day) in the first week of May 2007 among 101 selected household members in Røros**. AR = attack rate (%); n = total amount of members in this category.

Among the 101 selected household members, the risk of illness increased with the amount of daily consumed tap water (χ^2 ^for linear trend = 8.1; p = 0.004, Figure 3). No other common exposures were associated with gastrointestinal disease. A total of 23 selected household members (23%) reported noticing water abnormalities, at different days and hours during the study time.

### Retrospective study of institutions

Out of 11 interviewed institutions, only one kindergarten did not report gastroenteritis symptoms occurring among personnel and children in care. This kindergarten obtains water from the RH zone. Institutions with the highest AR obtain water from RL (Table 3).

**Table 3:** Attack rates (AR) in interviewed institutions in Røros (n = 11)

**Waterworks zone**	**Institution**	**Number of personnel**	Number of others*	**AR % staff**	AR % others*	**AR % total**
RH	kindergarten 1	2	14	0	0	0
	care centre 1	17	16	6	6	6
	nursing home	70	60	14	10	12

RL	care centre 2	60	28	8	7	8
	hospital	95	24	13	0	10
	rehabilitation centre	61	60	8	12	10
	kindergarten 2	21	76	19	16	16
	kindergarten 3	13	44	0	23	18
	kindergarten 4	12	47	17	23	22
	after school care	17	145	35	21	22
	kindergarten 5	5	20	40	45	44

AR: attack rate, RH: higher zone, RL: lower zone of Røros waterworks

* others = residents or hospitalized or children

### Microbiological analyses of patients' stool samples

The laboratory of medical microbiology at St Olavs Hospital received 171 stool samples from patients visiting Røros and Holtålen municipal health centres from 1 May to 30 June 2007. Counting only the first sample per patient, we received 62 fecal samples. Out of 61 samples cultured for *Campylobacter*, 32 were positive. Of these, three came from persons living in Holtålen with known exposure to tap water in Røros. Of the 32 *Campylobacter *isolates 21 isolates were analysed for genetic similarity by AFLP together with five additional isolates from patients in other municipalities, who had only visited Røros. Out of 26 tested in total, 25 strains had identical AFLP profile, including the three strains from Holtålen. The strain with a different profile belonged to a patient who had travelled abroad shortly before the illness.

Out of 38 samples cultured for *Salmonella, Shigella *and *Yersinia*, 37 samples were negative and one was positive for *Yersinia *and negative for *Campylobacter*. Among 35 samples tested for adenovirus, rotavirus and astrovirus, only one sample was positive for viral antigen (adenovirus 41) and its culture showed no growth of *Campylobacter*. All 38 samples tested for norovirus were negative.

Of eight samples tested for *Clostridium difficile *toxin A/B, one was toxin positive and negative for *Campylobacter*.

Thirty two specimens were examined for *Giardia lamblia *and all were negative. Microscopy of 18 available samples at the Norwegian School of Veterinary Science revealed two samples with *Cryptosporidium parvum *and one with *Giardia lamblia*. Out of these 18 samples, 15 were positive for *Campylobacter jejuni*, including the one sample positive for *Giardia*.

### Environmental investigation

The main part of Røros waterworks was constructed in 1979. The piping is of variable age, with some parts dating from 1910, and of variable materials. The supply to the lower area of Røros starts with 300 m of wooden main pipes from 1942. Considerable amount of leakage from Røros waterworks was reported in 2006 to the Waterworks registry at NIPH: 40% of the water was lost before reaching the customers [21].

Both wells and the common collecting tank are well protected. Our investigation established it would be possible for birds to enter through the ventilation of the elevated reservoir (Figure 1) and this opening was immediately secured.

With the exception of two occasions in 2005 and 2006 with samples positive for coliform bacteria (not *E.coli*), routine weekly water samples were satisfactory in the years preceding the outbreak.

The water samples, routinely taken on 24 April, were satisfactory (Table 4). Due to labour free day, the routine water samples were not taken on 1 May. The routine water samples from 8 May were additionally tested for faecal contamination. In these samples, no *E.coli*, other coliform bacteria or enterococci were isolated. Samples taken on 11 May were analysed for *Campylobacter spp*. and were negative. One single sample, taken at the main water intake at Røros dairy on 9 May was positive for coliform bacteria. Chlorination of water was started on the evening of 11 May.

**Table 4:** Water samples taken at different locations of Røros waterworks from 24 April to 15 May 2007

**Date**	**Sample type**	**Sampling site^#^**	**Tested for**	**Result**
24 April	Weekly routine	1,2,3,4	as described*	Satisfactory
1 May	Weekly routine	National holiday		No sample taken
8 May	Quarterly routine	4, 5, 6, 7	as described and: pH	Satisfactory
			Conductivity at 25°C	Satisfactory
			Intestinal enterococci	Satisfactory
9 May	Quarterly routine	Dairy main intake	Coliform bacteria	Coliform proven
			Total count at 22°C	High CFU in all tap samples
			*Cl. perfringens*	Satisfactory
			Intestinal enterococci	Satisfactory
11 May	Investigation	1,2,5	Coliform bacteria	Satisfactory
			*Campylobacter*	Satisfactory
11 May	Investigation	Slaughterhouse	Coliform bacteria	Satisfactory
			Total count at 22°C	Satisfactory
			Intestinal enteroccoci	Satisfactory
			*Cl. perfringens*	Satisfactory
11 May	Investigation	6	Coliform bacteria	Satisfactory
			Total count at 22°C	Satisfactory
15 May	Weekly routine	1,2,3,4	as described	Satisfactory
22 May	Weekly routine	4,5,6,7	as described	Satisfactory

^# ^1 municipal workers room, 2 airport, 3 hotel, 4 waterworks pump station, 5 fire station, 6 dairy, 7 nursing home

* As described in the Methods section

During the relevant period, some maintenance work was done in the distribution system: On 30 April, a broken waterworks' pipe was changed. During this procedure, the main pipe was closed and the pressure was not maintained, however there were no sewage pipes located parallel to the water pipes in this area. One of the main valves that help maintaining the pressure in the system was found closed in the vicinity of this work (Figure 1). The other maintenance was done on 2 May (Figure. 1) when only a smaller branch of the piping was closed. In both incidents, waterworks personnel did not follow the written procedures recommending chlorination after work on the distribution system.

The potential sources of *Campylobacter *contamination were difficult to pinpoint. Røros slaughterhouse, which mainly slaughters cattle and occasionally small ruminants, reported low water pressure at midday on 3 and 10 May. On 3 and 10 May, firemen performed an exercise testing the water output at the airport (RL zone) with more than 1000 L/min (Figure 1). The nearby Røros dairy also reported observing a pressure fall, but exact time was not confirmed. Plumbing at the dairy was routinely checked shortly before the outbreak and no failures were identified. The slaughterhouse is based in one of the lowest points of the waterworks and was not visited during the outbreak. Both slaughterhouse and dairy are situated in the RL zone and also in the vicinity of the first maintenance work.

With the exception of a few goats and hens, there is no livestock in the centre of Røros. Flocks of pigeons are frequently seen in front of the Røros city hall. In early May 2007, the lake that surrounds the underground wells was still covered with ice, thus preventing birds, animals and humans from access. At the time of the outbreak, water and melting snow were on the streets of Røros centre.

## Discussion

Our investigation shows that the large outbreak of gastrointestinal illness affecting about 4 out of 10 of the Røros waterworks customers (about 1500 people) was caused by tap water, contaminated with *Campylobacter*. We found a strong association between the amount of tap water consumed and illness. Although the cause of the water contamination was not identified, the identical genetic profiles of *C.jejuni *isolated from patients indicate a common source of the outbreak.

This outbreak is among the largest waterborne outbreaks reported in Norway [7,18]. In outbreaks caused by *Campylobacter*, late complications of the infection such as reactive arthritis arthritis [19] and Guillain-Barré syndrome [22] may add to the final disease burden. After this outbreak, one patient with arthritis was reported and according to the data, available at the time of writing this article, no patients with Guillain-Barré syndrome were detected.

Detailed data on response rate in our cohort study are missing, but we estimate less than 10% of the people contacted declined a phone interview. The epidemic curve (Figure 2) and the results of the AFLP analysis of *Campylobacter *positive samples suggest a common source outbreak. Three out of 18 samples tested for parasites were positive, therefore sewage contamination, which usually causes outbreaks with several pathogens [2] cannot be completely excluded. However, as the majority of isolated *Campylobacter *strains were identical, we believe other detected pathogens in the stool samples represent sporadic cases and the contamination was most likely caused by a livestock source or bird excrements rather than sewage.

Since the average incubation period for *Campylobacter *infection is 2–5 days [23], the most probable time of infection was around 1 May 2007. Person to person transmission of *Campylobacter *is uncommon [23] and since some cases fell ill after 11 May, this might be due to a longer incubation period or prolonged water contamination. In the months with continuous chlorination following the outbreak, no further cases were reported.

We were not able to demonstrate a difference in attack rates between the higher and the lower area of Røros, even with the help of the additional study among institutions. Since the daily activities of most people in the RH zone include working, shopping or attending school in the RL zone, a possible difference would be difficult to detect.

*Campylobacter *was not isolated from Røros waterworks samples taken on 11 May – a little more than one week after the probable contamination event. This bacteria can be difficult to detect in water samples, even if a contamination is strongly suspected [3,10,24]. The bacteria might be unevenly distributed in the waterworks system and the contamination might last only for a short period of time. In addition, the infectious dose is low and may be only a few hundred bacteria [9].

A previous retrospective community based survey using self reported illness to estimate the size of waterborne outbreaks has been criticized by Hunter and Syed [25]. The authors reported similar or higher attack rates in control towns, which did not receive contaminated water. They suspected the inhabitants in the control town learned about water contamination from the media and were more eager to report the illness and tap water consumption, even though they did not receive the water from the contaminated waterworks. Similarly, the media revealed drinking water as the suspected source in the Røros outbreak, thus the estimated size of this outbreak and the reported tap water consumption might have been biased. In Holtålen, the control town in our study, the attack rate was not high, but the inhabitants could have been aware their tap water came from a different source than Røros water. The exact recall period in Hunter's study is not reported and might have been longer than in our study (two weeks).

Waterborne outbreaks have been shown to result from several concurrent faulty factors. To ensure good drinking water risk management, critical barriers such as water treatment, source protection, distribution security and monitoring/response capabilities are needed [26]. The groundwater source in Røros was well protected and procedures for sampling and monitoring were established. However, multiple faults were discovered affecting the distribution system security: pressure fall, significant and persistent leakage problem, diverse and outdated materials of piping, including 300 m of wooden pipes, supplying the city centre.

Wood, used as a piping material, enables the aggregation of microorganisms, which form "biofilms". *Campylobacter *has not been shown to be an important component in biofilms [27], although that might depend on the bacterial strain [28].

Leakages in the system represent an increased risk of gastrointestinal illness among waterworks customers [29]. Low pressure incidents can cause intrusion of the microorganisms in the waterworks from the soil [29]. We identified two events in the lower area of Røros that led to a pressure fall during the relevant time. The maintenance work on 30 April, when pressure was not maintained, was done in vicinity of the slaughterhouse and dairy, where there might be potential sources of *Campylobacter *(raw milk, excrements). A recent study from South-western Norway has shown a 26% prevalence of *C. jejuni *in Norwegian cattle [30]. Standing water from melted snow at this time of the year could also be a source of contamination [24].

Routine chlorination is required when waterworks personnel conduct maintenance work. We recommend additional water samples to be taken in such cases to make sure the water quality is not affected. Improved training of waterworks personnel might lower the risk of negligence in the future.

In Røros, additional effort needs to be made to diminish the leakage problem, which makes the system vulnerable to pressure fall and contamination. Regular monitoring of the pressure has now been established. The wooden piping has been scheduled to be changed with cast iron piping in the summer of 2008. All fire valves were secured with lids to prevent contamination. Further plans to upgrade the system have been made. The firemen were advised to change their weekly emergency practice to allow more time for filling their tanks to avoid sudden pressure drop in the system.

Drinking water safety is largely taken for granted in affluent countries [26] and a strong belief in "naturally safe" water is common in Scandinavia [27]. Frequent waterborne outbreaks with severe social and economic burden in Norway [7] raise doubts about the adequacy of drinking water regulations. Our investigation has discovered weak points in the waterworks distribution system and its drinking water risk management. While untreated groundwater might be a safe water source, the distribution system is still vulnerable to peripheral contamination. This risk needs to be better acknowledged and managed.

## Conclusion

We describe a large waterborne outbreak of gastrointestinal disease in Røros traced to a waterworks with well protected groundwater source and with established regular water sampling and monitoring. Our investigation discovered several faults in the distribution system, which challenged the distribution security. Similar waterborne outbreaks in Scandinavia [10] with large public health impact suggest drinking water is vulnerable to peripheral contamination in the distribution system. A focus on improved distribution safety should be made, including stricter regulations, regular upgrading of the distribution system, thorough leakage investigation, chlorination related to maintenance work in the distribution system and pressure monitoring to identify low-pressure episodes. Waterworks personnel should provide a regular feedback to the authorities and should be trained to ensure good response capabilities in case of detected faults.

## Competing interests

The authors declare that they have no competing interests.

## Authors' contributions

IJ participated in the study design, data gathering, data entering and analysis and drafted the manuscript; KB participated in study design, data gathering and analysis, communication with local authorities and drafting of the manuscript, LV participated in study design, data gathering and communication with laboratory and local authorities, HL was leading the local investigation and participated in data gathering, TF participated in the environmental investigations and communicated with the local press, RH evaluated and reported the results of the laboratory analyses for local and national authorities and participated in drafting the manuscript regarding microbiology, KN participated in the study design, carried out the statistical analysis and drafting of the manuscript.

## Pre-publication history

The pre-publication history for this paper can be accessed here:


